# Pharmacodynamic and pharmacokinetic assessment of electronic cigarettes, combustible cigarettes, and nicotine gum: implications for abuse liability

**DOI:** 10.1007/s00213-017-4665-y

**Published:** 2017-06-20

**Authors:** Mitchell F. Stiles, Leanne R. Campbell, Donald W. Graff, Bobbette A. Jones, Reginald V. Fant, Jack E. Henningfield

**Affiliations:** 1 RAI Services Company, 401 N. Main Street, Winston-Salem, NC 27101 USA; 2 Celerion, Lincoln, NE USA; 3 Pinney Associates, Inc., Bethesda, MD USA

**Keywords:** Abuse liability, Electronic cigarettes, Nicotine pharmacokinetics, Pharmacodynamics, Product liking, Subjective measures

## Abstract

**Rationale:**

Electronic cigarettes (ECs) are becoming popular alternatives for smokers, but there has been limited study of their abuse liability.

**Objectives:**

The objective of this study was to evaluate the abuse liability of three Vuse Solo ECs, ranging from 14 to 36 mg in nicotine content, relative to high- and low-abuse liability comparator products (usual brand combustible cigarettes and nicotine gum, respectively) in a group of 45 EC-naïve smokers.

**Methods:**

Enrolled subjects’ ratings of subjective effects and nicotine uptake over 6 h were used to measure abuse liability and pharmacokinetics following in-clinic use of each EC.

**Results:**

Use of Vuse Solo resulted in subjective measures and nicotine uptake that were between those of combustible cigarettes and nicotine gum, although generally closer to nicotine gum. Compared to combustible cigarettes, use of Vuse Solo resulted in significantly lower scores in measures of product liking, positive effects, and intent to use again. These pharmacodynamic findings were consistent with the pharmacokinetic data, showing that cigarettes produced substantially faster and higher levels of nicotine uptake as compared to Vuse Solo and nicotine gum. Vuse Solo resulted in more rapid initial uptake of nicotine compared to nicotine gum, but peak concentration and long-term extent of uptake were not different or were lower with Vuse.

**Conclusions:**

Collectively, these findings suggest that Vuse Solo likely has an abuse liability that is somewhat greater than nicotine gum but lower than cigarettes.

**Trial registration:**

ClinicalTrials.gov identifier: NCT02269514

**Electronic supplementary material:**

The online version of this article (doi:10.1007/s00213-017-4665-y) contains supplementary material, which is available to authorized users.

## Introduction

A growing body of evidence supports the contention that electronic cigarettes (ECs) are a substantially less harmful alternative to combustible cigarettes for smokers (Caponnetto et al. 2012; Etter and Bullen 2013; Goniewicz et al. 2014; Farsalinos and Polosa 2014; Hajek et al. 2014; Hecht et al. 2015; McNeill et al. 2015; Truth Initiative 2015; Rass et al. 2015). Research suggests the existence of a pronounced “continuum of risk” of tobacco and nicotine products (Kozlowski et al. 2001; Zeller and Hatsukami 2009; O’Connor 2012; Hatsukami 2013; Nutt et al. 2014; U. S. Department of Health and Human Services 2014; Rass et al. 2015). At one end of the continuum, cigarette smoking poses the most significant risk of serious diseases. At the other end of the continuum, medicinal nicotine replacement therapies provide primarily nicotine and carry a very low risk of abuse, addiction, and harm (Murray et al. 1996; Waldum et al. 1996; Benowitz 2009; Murray et al. 2009; Cone et al. 2012; Newhouse et al. 2012; U. S. Department of Health and Human Services 2014). Similarly, nicotine-delivering products and dosage forms vary widely in their abuse liability or dependence potential, just as do products and dosage forms that contain other substances with a potential for abuse and dependence (Fagerström and Eissenberg 2012; Grudzinskas et al. 2006; Henningfield and Keenan 1993; US DHHS 2010; FDA 2010, 2015, 2017).

Between these two extremes are products that do not burn or contain tobacco, including ECs. ECs do not burn tobacco and operate at far lower temperatures than occur in the burning cigarette. Further, the chemistry of the EC aerosol has been well characterized in several laboratories as containing far fewer known toxicants, and those that have been measured are generally at far lower levels than are typical of cigarette smoke (Caponnetto et al. 2012; Etter and Bullen 2013; Goniewicz et al. 2014; Farsalinos and Polosa 2014; Hajek et al. 2014; Hecht et al. 2015). Thus, the consumer is exposed to far fewer toxicants and this has been widely predicted to carry much lower overall risks of disease, a conclusion advanced by Public Health England (McNeill et al. 2015), The Truth Initiative (2015), and other organizations and experts (Abrams and Niaura 2015; US DHHS 2014). Similarly, epidemiological evidence and preliminary clinical research suggest that ECs carry a lower risk of abuse than combustible cigarettes (Truth Initiative 2015; Vansickel et al. 2010; Vansickel et al. 2012b; Rass et al. 2015).

The Family Smoking Prevention and Tobacco Control Act of 2009 requires the FDA to consider the public health impact in the evaluation of new tobacco products, modified risk tobacco products, and in setting product standards for tobacco products. Public health concerns include the risk that the product will increase initiation, foster dependence, or interfere with cessation (US DHHS 2010; US DHHS 2012; The Family Smoking Prevention and Tobacco Control Act 2009). Assessment of abuse liability can help to address these concerns. Abuse liability has been defined as “the likelihood that individuals will engage in persistent and problematic use” of a drug and “the likelihood that individuals will experience undesirable consequences as a result of its use” (Carter et al. 2009; Calderon and Klein 2014). Abuse liability assessment is required by FDA for most drugs that affect the central nervous system (CNS) or are chemically or pharmacologically similar to drugs with known abuse potential as a component of FDA’s assessment of product safety and benefits and labeling (Calderon and Klein 2014; FDA 2010, 2017; The Expert Panel 2003; Schuster et al. 2003). For most areas of medicinal development involving CNS-acting drugs, the target product profile includes abuse liability that is so low as to avoid the need for labeling and warnings, or a requirement for Controlled Substance Act scheduling. However, for products developed to substitute for substances of abuse and/or assist in treatment of substance use disorders (e.g., buprenorphine), some level of abuse potential may be desirable to maintain compliance and support substitution in place of the substance of greater abuse potential and concern with greater potential for harm (Jones 2004; National Institute on Drug Abuse 2012; Substance Abuse and Mental Health Services Administration 2016).

Of all nicotine delivery products, cigarettes are considered to carry the highest adverse health effects and abuse liability or “addiction potential” due to their exceptionally rapid and efficient potential for nicotine absorption and transfer to the brain, as well as other substances in the smoke that may contribute to abuse liability (Royal College of Physicians of London 2000; US DHHS 2010; US DHHS 2014). We might also consider a continuum of dependence or abuse liability, with the same high and low anchors (combustible cigarettes and nicotine replacement therapies, respectively) as the risk continuum (Fagerström and Eissenberg 2012). Thus, the 2014 Surgeon General’s report and various tobacco control experts have concluded that alternative nicotine delivery products may be useful and appropriate to benefit public health by delivering sufficient nicotine, and with sufficient appeal and abuse potential, to be adopted by current smokers in place of combustible cigarettes (Abrams 2014; Hatsukami 2013; Shihadeh and Eissenberg 2015; Warner et al. 1997; Zeller 2013; Niaura 2016).

The current study was performed to examine elements of the abuse liability of three Vuse Solo ECs relative to high and low abuse liability comparator products (combustible cigarettes and nicotine gum, respectively) in current smokers. Elements of abuse liability assessed included a number of subjective measures and physiological effects, along with measures of the speed and amount of nicotine uptake following a single use of each study product.

## Materials and methods

### Subjects

Eligibility criteria were assessed during a screening process to ensure that subjects were in generally good health, satisfied all requirements for inclusion, and met none of the requirements for exclusion. General health evaluations included a standard physical and oral examination (including vital signs and an electrocardiogram), medical history (including concomitant medications), and clinical laboratory assessments (chemistry, hematology, urinalysis, virology, drug, and alcohol screening). Subjects were required to be 21 to 60 years of age, smoke 10 or more non-menthol 83 mm (king size) to 100 mm combustible filtered cigarettes per day for at least 6 months, and typically smoke their first cigarette of the day within 30 min of waking. Smoking behavior was self-reported during screening and subjects were required to have an expired breath carbon monoxide level ≥ 15 ppm to continue. An attempt was made to enroll an approximate balance of males and females. Primary exclusion criteria included any clinically significant medical condition that would preclude the subject from participating in the study, postponement of a smoking quit attempt to participate in the study, use of any smoking cessation product within 30 days of screening, use of any tobacco or nicotine-containing product (including ECs) other than combustible cigarettes within 30 days of screening, and females who were pregnant or lactating.

### Investigational products

Three, non-menthol, commercially available brand styles of Vuse Solo were evaluated in this study, containing either 14, 29, or 36 mg of nicotine. Vuse Solo ECs are composed of a battery, heating element, microchips, sensor, and a cartridge containing propylene glycol, glycerin, nicotine, flavorings, and water. The three ECs were presented without brand style information and were visually indistinguishable by subjects.

Usual brand cigarettes (any combustible, filtered, non-menthol brand style, 83 mm [king size] to 100 mm in length) and Nicorette® White Ice Mint nicotine polacrilex gum, 4 mg (GlaxoSmithKline Consumer Healthcare, L.P.) were chosen as high and low abuse liability comparator products, respectively, to assess the relative abuse liability of Vuse Solo (Stitzer and de Wit 1998; West et al. 2000; Houstsmuller et al. 2002; Johnson and Bickel 2003; Johnson et al. 2004). Positive and negative controls are discussed in the 2010 Assessment of Abuse Potential of Drugs (FDA 2010, 2017) guidance when designing studies to evaluate abuse liability. In this study, the high comparator product (i.e., combustible cigarette) and low comparator product (i.e., nicotine gum) are basically equivalent to the positive and negative control, respectively, and are therefore consistent with FDA guidance in the assessment of Vuse Solo. The three Vuse Solo ECs and nicotine gum were provided at no cost to subjects, while subjects provided their own usual brand cigarettes throughout the study.

### Study design

This was a randomized, open-label, cross-over study (ClinicalTrials.gov identifier: NCT02269514) completed at a single research center (Celerion, Lincoln, NE). The study was reviewed and approved by Chesapeake Institutional Review Board (Columbia, MD) and was conducted in accordance with the ethical standards in the Declaration of Helsinki and applicable sections of the United States Code of Federal Regulations and ICH E6 Good Clinical Practices. Study candidates were recruited using standard advertising methods (print, radio, television) and from an existing database of individuals who had previously participated, or who previously expressed interest in participating, in a clinical study. Informed consent was obtained from all potential subjects prior to initiation of any study events.

### General methods

#### Ambulatory periods

Eligible subjects who successfully passed all screening requirements were enrolled into the study and randomized to a product use sequence. A 7-day ambulatory (“home use”) trial of each investigational product (including a week of using only usual brand cigarette) preceded each of five test visits to allow subjects to become accustomed to using the new products. Instructions for product use (Vuse ECs and nicotine gum) were provided by study staff upon dispensation for the at-home trial periods. Specific instructions relevant to use of Vuse Solo ECs (e.g., changing spent cartridges, recharging the battery, meaning of LED indicator lights) were demonstrated and/or communicated to subjects. Product use during the ambulatory periods was non-exclusive, as subjects were allowed to smoke their usual brand cigarettes throughout the study. Product use was tracked daily using an electronic diary, with subjects documenting the number of usual brand cigarettes smoked and the number of “uses” of Vuse Solo or nicotine gum per day (data not presented). One “use” of Vuse Solo or nicotine gum was defined as approximately 10 to 30 min of ad libitum use, respectively, to approximate use in test visits.

Subjects were instructed to use the assigned investigational product at least once per day for 6 of the 7 days prior to each test visit; subjects were not to use the dispensed investigational products on the day immediately prior to the test visit. Use of usual brand cigarettes during each day of the at-home trial was allowed regardless of the investigational product assignment. Subjects were to abstain from all tobacco and nicotine products for at least 12 h prior to each test visit to minimize the impact that residual nicotine concentrations might have on baseline subjective and physiological measurements.

#### Test visits

Subjects reported to the clinic on the morning of each test visit and were initially assessed for continued eligibility and compliance with the required 12-h smoking abstention. Subjects with an expired carbon monoxide value >12 ppm were not eligible to participate in the clinical procedures on that day but were allowed to reschedule one test visit for this reason. In-clinic product use, all ad libitum, consisted of up to 10 min use of Vuse Solo or smoking of one cigarette, or up to 30 min using nicotine gum according to the package instructions (i.e., “park and chew” method). Serial blood sampling, questionnaires, and physiological measurements were completed at the specified time points relative to the start of product use (see Supplementary Table 1).

Individual Vuse Solo cartridge weights, before (initial weight) and after (final weight) in-clinic use, were recorded to assess the amount of product use. In-clinic use of each of the three types of products occurred in separate sections of the clinic to minimize any potential effects of environmental aerosol or tobacco smoke or other sensory cues on subjective effects assessments. Subjects underwent End-of-Study procedures at test visit 5 (or early termination), including a symptom-driven physical examination, a brief oral examination, and collection of blood and urine samples for clinical laboratory tests.

#### Subjective measures

Five different questionnaires were administered to assess subjective endpoints: Product Liking, Intent to Use Product Again, Product Effects, Urge to Smoke, and Urge for Product. The questionnaires were completed by the subjects using a tablet device (CRFHealth, Hammersmith, UK). The Product Liking (“How much did you like your [usual brand cigarette, electronic cigarette, nicotine gum]?”), Urge to Smoke (“How strong is your current urge to smoke your usual brand cigarette?”), and Urge for Product (“How strong is your current urge to [use your electronic cigarette/chew nicotine gum]?”) questionnaires were administered as 10-point numeric rating scales with “Did not like at all” or “No urge” as the left anchor and “Liked Extremely” or “Extremely Strong Urge” as the right anchor. The Intent to Use Product Again questionnaire (“I would choose/intend to use [my usual brand cigarette, my electronic cigarette, nicotine gum] again”) was administered as a 7-point vertical numeric scale ranging from “Strongly disagree” at the bottom (followed by “Disagree,” “Slightly disagree,” “Neither agree nor disagree,” “Slightly agree,” “Agree”) to “Strongly agree” at the top.

The Product Effects questionnaire was designed to assess positive and negative effects of product use in a step fashion based on previous response. The first question was administered in Yes/No format: “Do you feel any positive or negative effects of the product right now? This could include any type of effect including physical, mental, or other effects.” If the question was answered in the affirmative, a 5-point scale was used to assess whether the effects were perceived as positive, negative, or both. Any presence of positive or negative effects led to evaluation of the overall effects on a 10-point numeric rating scale (“Overall, how much do you like [or dislike] the positive [or negative] effects you are feeling now?”, anchored with “Like a little/Dislike a little” and “Like very much/Dislike very much”). Subjects were also asked to identify specific positive and negative effects from a list of the effects felt at the time (e.g., calm, able to concentrate, headache, nausea, cough).

#### Nicotine pharmacokinetics

A series of timed blood samples was collected for measurement of nicotine concentration to assess uptake from product use. Eighteen samples were drawn via single venous samplings in each of the five test visits (see Supplementary Table 1). Collection times were −5, −0.5, 3, 5, 7.5, 10, 15, 20, 30, 45, 60, 90, 120, 150, 180, 240, 300, and 360 min relative to the subject starting use of product.

#### Physiological measures

Physiological measures included pulse rate, systolic and diastolic blood pressure, and expired carbon monoxide. Baseline cotinine concentrations were also measured to assess whether subjects substantially changed their nicotine uptake during the study. Safety and tolerability were evaluated based on data collected from physical and oral examinations, clinical laboratory tests, vital sign measurements, electrocardiograms, and adverse events.

#### Statistical analyses

Based on existing knowledge and the requirements of the randomization method (i.e., Williams Design), the target number for completion was 40 subjects in order to have 80% power for detecting the hypothesized differences between Vuse Solo and the usual brand cigarette. The hypothesized differences were an effect size of 0.8 for the subjective measurements (assuming a correlation of 0.6, which is equivalent to a mean difference of 0.8 and a standard deviation of 1.0) and ±20% for the pharmacokinetic (PK) endpoints. Statistical significance is indicated for *p* values below 0.05.

Data management and statistical analyses were performed by Celerion (Lincoln, NE). Phoenix® WinNonlin® Version 6.3 (Pharsight, Princeton, NJ) was used to calculate non-compartmental PK and subjective measure response parameters. Statistical summarizations and comparisons were calculated using SAS® Version 9.3 (SAS, Cary, NC).

A mixed-effect model analysis of variance (ANOVA) was used to compare the following: subjects’ product liking peak effect (*E*_max_) and area under the effect curve (AUEC)_15–360_; intent to use product again *E*_max_; (liking of) positive effects *E*_max_; and (disliking of) negative effects *E*_max_. Sequence, period, and product were included as fixed effects, and subject-nested-within-sequence was included as a random effect. All parameters were analyzed on the original scale. Additionally, for positive effects liking and negative effects disliking, a value of zero was assigned to any time points for which subjects responded as not feeling those effects on the initial Yes/No question (any positive or negative effects). A mixed-effect model analysis of covariance (ANCOVA) was used to compare urge to smoke AUC_0–15_, AUC_0–360_, *E*_min_, and *T*_min_; and urge for product *E*_max_ between each Vuse Solo EC and the usual brand cigarette and nicotine gum. Sequence, period, product, and the baseline score were included as fixed effects, and subject-nested-within-sequence was included as a random effect. All parameters were analyzed on the original scale. Since the Urge for Product questionnaire was not administered during usual brand cigarette use, the data collected from the Urge to Smoke questionnaire from the cigarette condition was compared to the Urge for Product data. The comparisons of interest were each of the Vuse Solo ECs to the respective comparator products; the three Vuse Solo ECs were not compared to one another.

Measured nicotine concentrations below the limit of quantitation (0.200 ng/ml) were set to one-half of the lower limit of quantitation for data summarization, statistical analysis, and calculation of the PK parameters. Concentrations measured from the post-baseline time points were adjusted for the concentration of nicotine in the blood at time 0 (i.e., the start of product use). Exponential decay expressed in terms of nicotine half-life was used, and the adjusted concentration was calculated as described by others (Shiffman et al. 2009; Benowitz et al. 2006). Any resulting negative concentration values following the baseline adjustment were set to 0.

A mixed-effect model analysis of variance (ANOVA) was used to compare the plasma nicotine PK parameters between each Vuse Solo EC and the usual brand cigarette and nicotine gum comparators. No comparisons were made among the three Vuse Solo ECs. Sequence, period, and product were included as fixed effects, and subject-nested-within sequence was included as a random effect. The AUC and *C*_max_ PK parameters were analyzed on the natural log scale, while *T*_max_ was analyzed on the original scale. Ratios and 90% confidence intervals for the ratios were calculated for the AUC and *C*_max_ parameters. A significant difference between two products was concluded if the true geometric mean ratios of AUC_nic 0–360_ or *C*_max_ for nicotine were less than 0.8 or greater than 1.25.

A mixed-effect model ANOVA was used to compare the maximum absolute change in physiological measures (i.e., pulse rate and systolic and diastolic blood pressures) between the high and low abuse liability comparators and each Vuse Solo. No comparisons were made among the three Vuse Solo ECs. Sequence, period, and IP were included as fixed effects, and subject-nested-within-sequence was included as a random effect. The change in expired carbon monoxide measurements from baseline was calculated per subject, and a paired *t*-test was performed to determine if the change value was significant.

## Results

### Subjects

One hundred twenty-one subjects took part in the screening procedures, 59 subjects were randomized, and 45 subjects completed all five test visits. Fourteen subjects were withdrawn from the study, including one subject who was discontinued due to adverse events (judged to be unrelated to study product), eight subjects who were discontinued due to protocol deviations, and five subjects who withdrew consent for study participation. Demographic data are shown in Table 1. A total of 30 different usual brand cigarette brand styles were reported as currently being smoked at the time of screening. Nearly half of the subjects reported smoking the four most common usual brand cigarette styles: Marlboro Red (*n* = 8, 14%), Marlboro Gold (*n* = 8, 14%), Pall Mall Red (*n* = 6, 10%), and Camel Blue (*n* = 5, 9%). Other brand styles were smoked by four or fewer (≤7%) subjects each. No subject reported regular use of ECs prior to entering the study.

**Table 1:** Demographic summary

Sex, *n* (%)	
Female	25 (42%)
Male	34 (58%)
Race, *n* (%)	
Asian	1 (2%)
White	56 (95%)
White, American Indian/Alaska Native	2 (3%)
Ethnicity	
Hispanic or Latino	3 (5%)
Not Hispanic or Latino	56 (95%)
Age, years	
Mean	39.7
SD	11.15
Body mass index, kg/m^2^	
Mean	27.1
SD	4.60
Cigarettes per day	
Mean	20.6
SD	6.34
Fagerström Test for Nicotine Dependence score	
Mean	5.8
SD	1.29

### Subjective measures

As illustrated in Table 2, the mean maximum scores (*E*_max_) on the Product Liking questionnaire were substantially lower for the three Vuse Solo ECs (LS [least square] mean *E*_max_ scores ranging from 4.13 to 4.57) compared to the cigarette condition (LS mean *E*_max_ = 9.06, *p* < 0.001 for all), and somewhat higher than nicotine gum (LS mean *E*_max_ = 3.21, *p* < 0.05 for all). A similar pattern was seen with the Intent to Use Again questionnaire. The mean *E*_max_ intent to use again scores were substantially lower for the three Vuse Solo ECs (LS mean *E*_max_ scores ranging from 4.07 to 4.75) compared to the cigarette condition (LS mean *E*_max_ = 6.81, *p* < 0.001 for all), and higher than nicotine gum (LS mean *E*_max_ = 3.29, *p* < 0.006 for all). A similar pattern was also shown for the Liking for Positive Effects measure. Among subjects who reported positive effects, mean *E*_max_ liking for positive effects scores were substantially lower for the three Vuse Solo ECs (LS mean *E*_max_ scores ranging from 5.99 to 6.71) compared to the cigarette condition (LS mean *E*_max_ = 8.31, *p* < 0.001 for all). Only the 14 and 29 mg Vuse Solo ECs were rated higher than nicotine gum (LS mean *E*_max_ = 5.47, *p* < 0.05 for both); the 36 mg Vuse Solo EC was not rated as significantly different from nicotine gum. Among those subjects who reported negative effects, there were no significant differences between any of the products on disliking for negative effects. Table 2 provides a summary of LS means for subjective measures. On the Urge for Product measure (only asked after use of Vuse Solo and nicotine gum), subject scores were higher with the three Vuse Solo ECs (LS mean *E*_max_ scores ranging from 4.15 to 4.58) compared to nicotine gum (LS mean *E*_max_ = 2.98, *p* < 0.005 for all).

**Table 2:** LS means of subjective measures

	LS means				
Parameter	Vuse Solo 14 mg	Vuse Solo 29 mg	Vuse Solo 36 mg	Usual brand cigarette	Nicotine gum
Product Liking (AUEC_15–360_)	1396.68*^, †^	1430.66*^, †^	1190.01*^, †^	3116.52	799.38
*E*_max_	4.36*^, †^	4.57*^, †^	4.13*^, †^	9.06	3.21
Intent to Use Again (AUEC_15–360_)	1619.43*^, †^	1635.82*^, †^	1400.99*^, †^	2369.30	1091.84
*E*_max_	4.71*^, †^	4.75*^, †^	4.07*^, †^	6.81	3.29
Liking of Positive Effects (AUEC_15–360_)	727.42	800.57*	673.67	889.74	444.17
*E*_max_	6.71*^, †^	6.51*^, †^	5.99*	8.31	5.47
Disliking of Negative Effects (AUEC_15–360_)	502.66	827.41	740.85	423.38	422.14
*E*_max_	6.03	6.41	6.67	5.80	6.28

*Significantly different from usual brand cigarette; *p* < 0.05

^†^Significantly different from nicotine gum; *p* < 0.05

### Urge to smoke

**Fig. 1:**
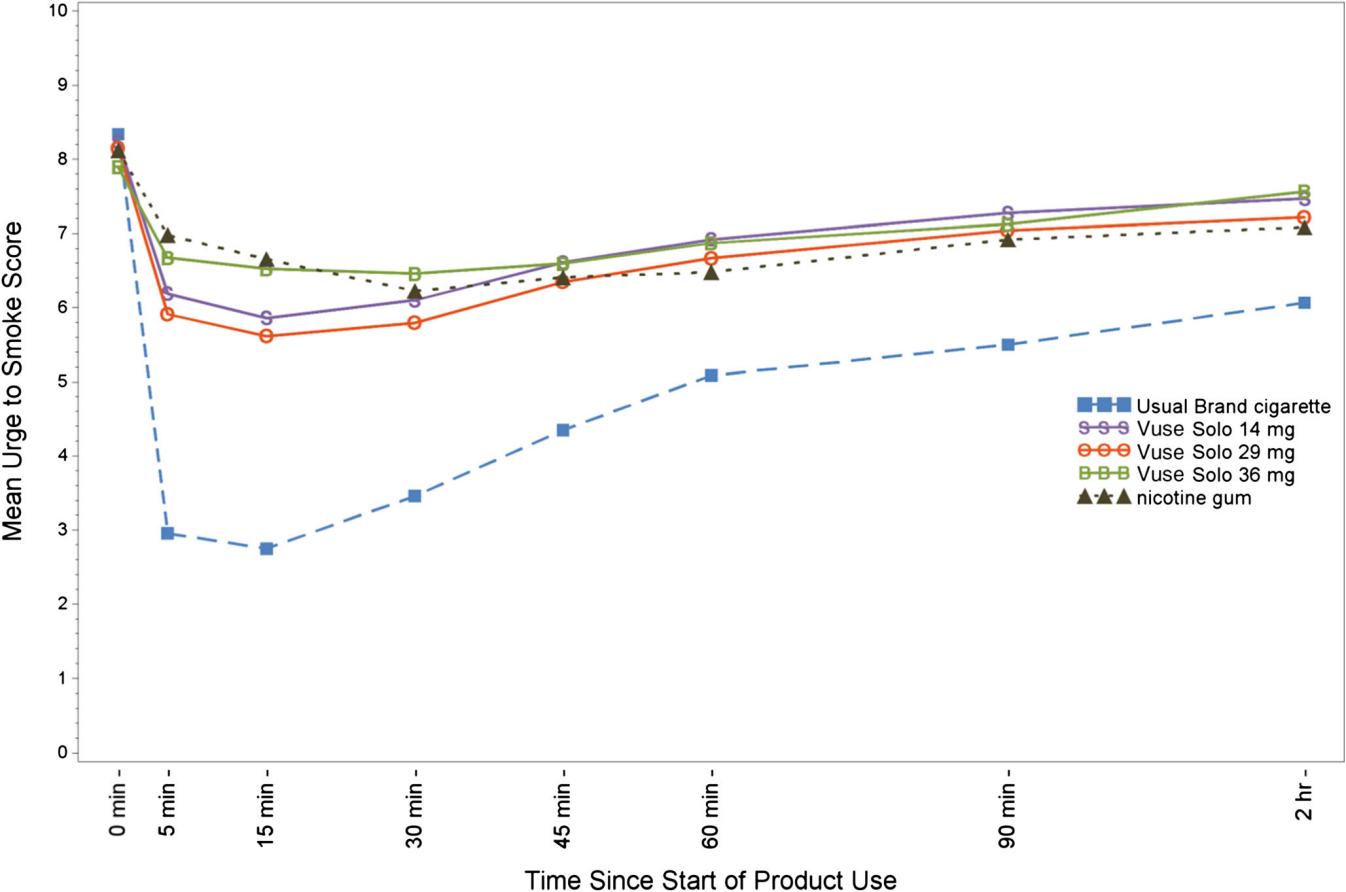
Mean ratings for the urge to smoke question “How strong is your current urge to smoke your usual brand cigarette?”

As illustrated in Fig. 1, within the first 15 min following start of product use, Urge to Smoke scores were lower with smoking (LS mean AUEC_0–15_ = 60.52) compared to Vuse Solo ECs (LS mean AUEC_0–15_ ranged from 94.52 to 104.38, *p* < 0.0001 for all). Urge to Smoke scores with nicotine gum (LS mean AUEC_0–15_ = 107.35) were significantly higher than Vuse Solo 14 mg (*p* < 0.05) and Vuse Solo 29 mg (*p* < 0.01), but not different from Vuse Solo 36 mg. Across the entire 6-h session, Urge to Smoke scores were lower with smoking (LS mean AUEC_0–360_ = 2290.86) compared to Vuse Solo ECs (LS mean AUEC_0–360_ ranged from 2715.24 to 2823.38, *p* < 0.0001 for all). Urge to Smoke scores with nicotine gum (LS mean AUEC_0–360_ = 2773.64) were not significantly different from any of the Vuse Solo ECs.

The time taken to reach the minimum urge to smoke (*T*_min_) was not significantly different between the three Vuse Solo ECs (LS mean *T*_min_ ranged from 17.95 to 24.73 min) and cigarettes (13.78 min). The *T*_min_ was reached significantly faster with use of the Vuse Solo 14 mg (LS mean *T*_min_ = 20.73 min) and Vuse Solo 29 mg (17.95 min) compared to nicotine gum (34.69 min) (*p* < 0.05 for both), but the *T*_min_ was not different between Vuse Solo 36 mg (24.73 min) and nicotine gum.

### Nicotine pharmacokinetics

**Fig. 2:**
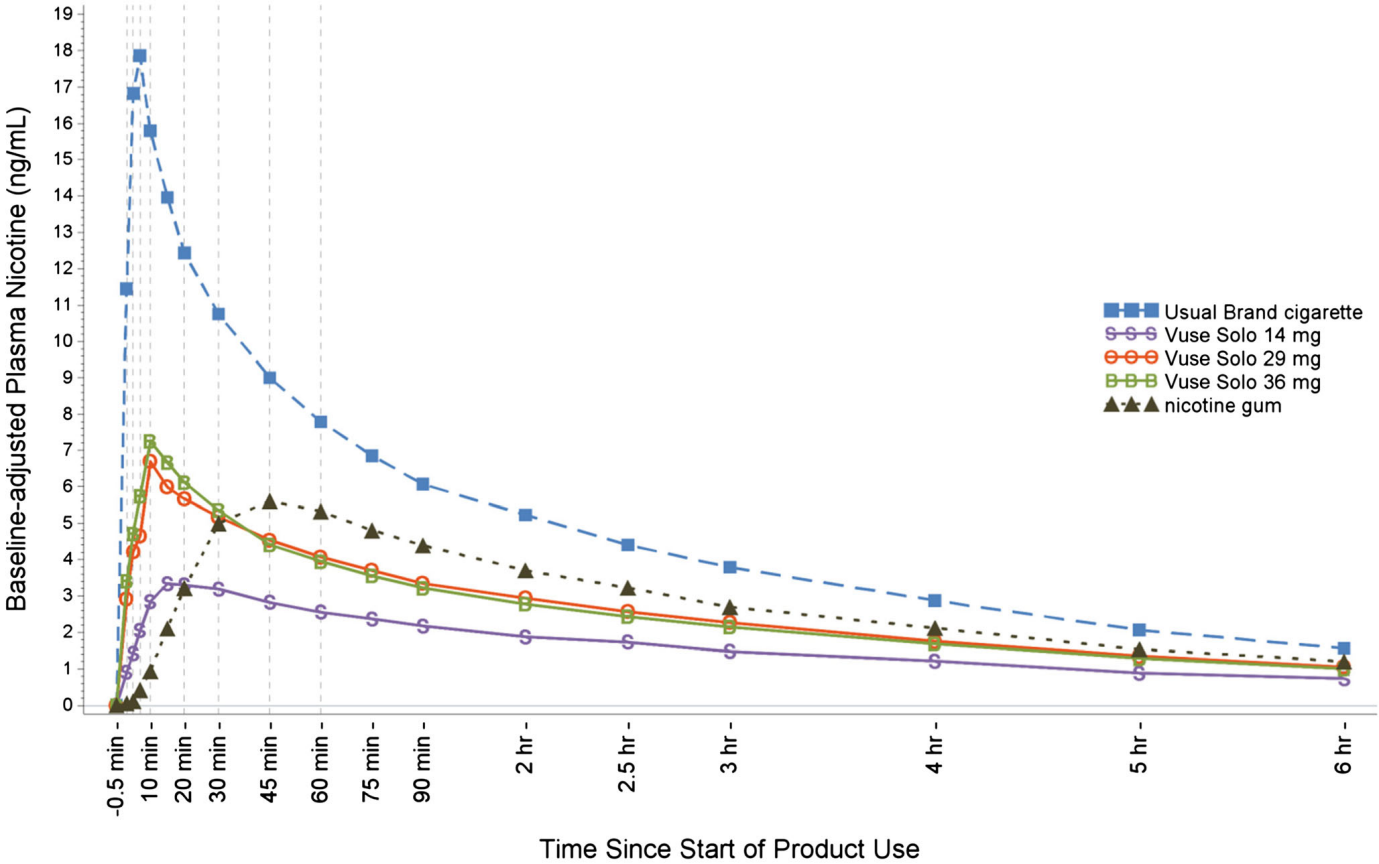
Mean plasma nicotine concentration profiles

Figure 2 illustrates the plasma nicotine curves for the study products. After 1 h, blood levels gradually declined to near convergence at about 2 ng/ml by 6 h. Table 3 summarizes the nicotine PK parameters. For the first 15 min following start of product use, plasma nicotine concentrations were significantly higher with smoking compared to the three Vuse Solo ECs (AUC_0–15_, *p* < 0.0001 for all), and all Vuse Solo EC results were significantly higher than with nicotine gum (AUC_0–15_, *p* < 0.0001 for all). Across the 6-h session, plasma nicotine concentrations were significantly higher with smoking compared to the three Vuse Solo ECs (AUC_0–360_, *p* < 0.0001 for all), and were significantly higher with nicotine gum compared to the three Vuse Solo ECs (AUC_0–360_, *p* < 0.003 for all). Similarly, *C*_max_ was significantly higher with smoking compared to use of Vuse Solo ECs (*p* < 0.0001 for all). However, there was no difference between the *C*_max_ with nicotine gum compared to Vuse Solo 29 mg and Vuse Solo 36 mg, while *C*_max_ was significantly lower with Vuse Solo 14 mg compared to nicotine gum (*p* < 0.0001).

**Table 3:** Statistical comparisons of baseline-adjusted plasma nicotine uptake parameters

	Geometric LS means^a^				
Parameter	Vuse Solo 14 mg	Vuse Solo 29 mg	Vuse Solo 36 mg	Usual brand cigarette	Nicotine gum
*C*_max_ (ng/ml)	3.01*^, †^	4.67*	5.36*	17.98	5.26
AUC_nic0–15_ (ng*min/ml)	22.30*^, †^	42.64*^, †^	37.30*^, †^	180.72	5.89
AUC_nic0–360_ (ng*min/ml)	482.39*^, †^	642.70*^, †^	658.97*^, †^	1670.32	884.22
*T*_max_ (minutes)	27.35*^, †^	21.83*^, †^	24.17*^, †^	8.13	50.88

*Significantly different from usual brand cigarette; *p* < 0.05

^†^Significantly different from nicotine gum; *p* < 0.05

^a^ *T*_max_ was analyzed on the original scale, thus arithmetic (i.e., not geometric) LS means are presented

### Baseline plasma cotinine concentrations

Plasma cotinine concentrations were measured in baseline samples collected at each test visit to assess whether subjects substantially changed their nicotine uptake during the study. Baseline LS means values for each study product ranged from 213.13 to 223.20 ng/ml and no differences were noted between any of the study products, indicating that the overall tobacco/nicotine product use did not change greatly throughout the study.

### Product use

The duration of use of the usual brand cigarettes and nicotine gum during the test visits was limited to ad libitum use of a single unit of product, for a maximum allowed time period of 10 and 30 min, respectively. The amount of ad libitum Vuse Solo use was evaluated via pre- and post-use cartridge weight differences during the maximum allowed time period of 10 min. Based on the mean difference in cartridge e-liquid weights, subjects tended to use more of Vuse Solo 14 mg (0.061 g), followed by Vuse Solo 29 mg (0.048 g) and Vuse Solo 36 mg (0.026 g).

### Physiological effects

Supplementary Table 2 summarizes the mean absolute changes in pulse rate and blood pressure following use of the Vuse Solo ECs, usual brand cigarette, and nicotine gum and the percent difference in change between Vuse Solo ECs and the other two conditions. Mean pulse rates at baseline were comparable prior to use of each of the study products, ranging from a mean of 62.8 to 66.4 bpm. There were no significant differences in absolute changes in pulse rate between the cigarette and Vuse Solo EC conditions. There were also no significant differences in the changes in pulse rate between the nicotine gum and the Vuse Solo 14 and 36 mg conditions; however, the change in pulse rate was slightly, but significantly greater with the Vuse Solo 29 mg compared to nicotine gum (*p* < 0.015) conditions.

There were some statistically significant, albeit small, differences in the changes in blood pressure with use of the Vuse Solo ECs versus the comparator products (see Supplementary Table 2). For systolic blood pressure, the only significant difference seen was a smaller increase after the Vuse Solo 14 mg compared to nicotine gum. For diastolic pressure, there was a significantly smaller increase after each of the three Vuse Solo ECs compared to both cigarettes and nicotine gum; however, the largest difference in change was only 4.12 mmHg, suggesting that these differences likely have little clinical significance.

### Expired carbon monoxide

Mean baseline expired carbon monoxide LS mean values were comparable prior to use of each study product, ranging from 6.44 to 7.58 ppm. As expected, the difference from baseline value was relatively unchanged following use of the three Vuse Solo ECs and nicotine gum (differences ranging from −0.39 to 0.39 ppm), but increased significantly following use of the usual brand cigarette (6.09 ppm, *p* < 0.0001).

### Safety

The study products were well-tolerated under the conditions of use during the study. A total of 95 adverse events were reported by 25 of the 59 subjects, roughly half of which were considered to be either related (2) or possibly related (42) to study product use. The vast majority (93) of the adverse events were mild in severity, while two (influenza and presyncope) were moderate. Headache was the most common adverse event reported during this study (15 episodes reported by 8 subjects), followed by nausea (11 episodes reported by 5 subjects), and cough (5 episodes by 5 subjects). One subject was discontinued due to adverse events associated with an unrelated illness. Fewer episodes of adverse events were reported with use of the three Vuse Solo ECs (8 to 14) than with the usual brand cigarette (23) or nicotine gum (43).

## Discussion

This study provides a comparative evaluation, based upon limited product exposure, of the pharmacodynamics and the pharmacokinetics of nicotine. The results are relevant to a comparative assessment of product abuse liability for ECs relative to known high and low abuse liability comparators (i.e., usual brand cigarettes and 4 mg nicotine gum, respectively). The results support the conclusion that the abuse liability for the Vuse Solo ECs tested in this study is substantially lower than that of combustible cigarettes, but higher than that of nicotine gum. This is consistent with the pharmacokinetics of the products: the cigarettes produced faster and higher nicotine uptake than that measured with Vuse Solo ECs, which was faster (but not higher) than that observed with use of nicotine gum. However, nicotine gum yielded higher overall nicotine uptake than even the highest nicotine content Vuse Solo EC. Taken together, the findings in this study extend earlier findings with nicotine and other dependence-producing substances that the abuse liability of a given substance can be strongly influenced by the form or formulation and route of administration and is generally related to the speed of absorption of the substance (FDA 2010, 2015, 2017; Calderon and Klein 2014; Fant et al. 1999; Fant et al. 1997; Grudzinskas et al. 2006; Henningfield and Keenan 1993; Reissig et al. 2015).

On most subjective measures, including the Product Liking, Intent to Use Again, and Liking of Positive Effects/Disliking of Negative Effects questionnaires, Vuse Solo ECs were rated intermediate to the usual brand cigarette and nicotine gum (Table 2). There was a modest but direct dose-response relationship across the three nicotine concentrations of Vuse Solo ECs, with generally little difference between the two highest concentrations (29 and 36 mg). That is, plasma nicotine values increased most rapidly and reached the highest values with cigarette smoking, and were slower and lower with Vuse Solo ECs and nicotine gum. Similarly, Product Liking scores reached the highest values with cigarette smoking, and produced the lowest values with nicotine gum use. Vuse Solo EC values were intermediate, with Product Liking scores being slightly, but significantly, greater than with nicotine gum, and much lower than with cigarette smoking.

Although studies to assess the speed and efficiency of nicotine uptake are presumed important indicators of the abuse liability of tobacco products by the Center for Tobacco Products (FDA 2012), speed of absorption is only one of multiple variables that are typically measured in prototypical pharmaceutical abuse liability studies (FDA 2017); other factors related to the product form sensory characteristics may influence abuse liability and measures of product liking (Carter et al. 2009; Hughes et al. 1984; Fagerström 2012; Calderon and Klein 2014; FDA 2015, 2017; Grudzinskas et al. 2006; Royal College of Physicians of London 2000; Henningfield et al. 2011). Thus, for abuse liability assessment, general subjective effects, and “drug liking” in particular, are the hallmark measures for comparison across substances, doses, and formulations (Calderon and Klein 2014; Carter et al. 2009; FDA 2010, 2015, 2017; The Expert Panel 2003). Well-researched, standard methodology exists for the study of abuse liability of pharmaceutical products, much of which is potentially transferrable to the assessment of abuse liability of new and existing tobacco products (Carter et al. 2009; FDA 2010, 2017). The speed and efficiency of nicotine uptake following tobacco product use can be studied in a manner similar to PK studies of pharmaceuticals. Similarly, assessment of subjective measures of product liking, intent to use the product again, and product effects (both positive and negative) used in this study are modifications of standard subjective measures for pharmaceutical compounds and may be useful in the evaluation of tobacco products.

Subjective ratings of drug liking in abuse liability studies of pharmaceuticals tend to be one of the most frequently used, most sensitive, and most reliable measures of likelihood of repeated use (The Expert Panel 2003; Carter et al. 2009; FDA 2010, 2017; Calderon and Klein 2014). Other measures that generally co-vary with drug liking include ratings of good effects (directly), bad effects (inversely), and the degree to which someone says they would take the drug again (directly) (Carter et al. 2009). In this study, we included measures of product liking, intent to use the product again, positive effects, and negative effects as additional factors that may contribute to the potential for product adoption. Liking for positive effects and disliking for negative effects were assessed with a modification of the Drug Any Effect/Drug Liking/Drug Disliking methods used in prototypical pharmaceutical abuse liability studies (McColl and Sellers 2006).

The subjects enrolled in this study were established smokers who were naïve to use of ECs; therefore, a preference toward the usual brand cigarette was expected and indeed was observed. This is a strength of the study and adapts the positive control strategy used in pharmaceutical abuse liability studies.

Results from the product liking, intent to use again, and positive product effects assessments used in this study were found to be higher for the usual brand cigarette compared to the three Vuse Solo ECs. Further, the subjective responses observed with Vuse Solo ECs were generally more similar to those produced by nicotine gum than by usual brand cigarette. The strongest points of difference between Vuse Solo ECs and nicotine gum appear to be a stronger intent to use Vuse Solo ECs again and the urge for product. Overall, these data indicate that the abuse liability of Vuse Solo ECs evaluated in the current study appears substantially lower than for cigarettes, as has been found with ECs in other studies (Vansickel and Eissenberg 2012a; Vansickel et al. 2012b).

The maximum nicotine concentrations reached with each of the products in the current study were generally consistent with concentrations measured at similar time points in previous studies. Compared to the usual brand cigarette, the rate of nicotine uptake was slower and overall uptake was lower with each of the Vuse Solo ECs. Further, though the maximal concentrations reached were no different or were lower with Vuse Solo ECs compared to the nicotine gum, uptake from Vuse Solo ECs was more rapid and higher over the first 15 min of use, but was lower for the entire 6-h sampling period. These findings are not unexpected based on the route of administration and the duration of use. Inhaled nicotine is rapidly dispersed throughout the circulatory system, with peak concentrations in the blood reached within minutes of completion of use, as was observed here, compared to buccal absorption. Although the duration of smoking episodes was technically limited in the current study (up to 10 min), a single cigarette is typically consumed in as few as 5 to 7 min, and use of Vuse Solo ECs was also allowed over a 10-min period. In both cases, maximal concentrations were reached soon after product use was completed, more quickly for the usual brand cigarette. In contrast, uptake from the nicotine gum continued for a longer period of time after product use was complete.

The minimum urge to smoke score and the time to minimum urge to smoke followed a pattern similar to nicotine uptake, though the relative differences in the urge to smoke parameters between the three Vuse Solo ECs and the comparator products were not as extreme as the corresponding nicotine uptake parameters, an observation consistent with the possible contribution of certain ritual behavioral components of smoking in relieving smoking urges. Not surprisingly, urge to smoke was significantly lower overall with the usual brand cigarette compared to Vuse Solo ECs. Further, whereas some reports suggest that ECs may be more efficacious than traditional nicotine replacement therapies for smoking cessation (Barbeau et al. 2013), despite providing relatively low levels of nicotine compared to combustible cigarettes (Vansickel and Eissenberg 2012a; Vansickel et al. 2012b), we found few significant differences between Vuse Solo ECs and nicotine gum among the urge to smoke parameters evaluated in this study. For example, urge to smoke scores with nicotine gum were significantly higher than Vuse Solo 14 mg and Vuse Solo 29 mg, but not different from Vuse Solo 36 mg. However, one purpose of the current study was to compare the ability of Vuse Solo ECs to satisfy urge to smoke over the short term, and a single product use is not sufficient to evaluate the potential utility of a product for smoking cessation.

Physiological effects are often included in abuse liability studies because the measures are objective and provide potential physiological correlates of subjective effects (Carter et al. 2009). Nicotine is known to increase heart rate and blood pressure with acute administration from tobacco and other nicotine-containing products (Benowitz et al. 2002; Yan and D’Ruiz 2015). This is consistent with our observations, though the changes from baseline in pulse rate and systolic and diastolic blood pressure between Vuse Solo ECs and both comparator products were generally comparable. Also evaluated, and as expected, a significant increase from baseline in expired carbon monoxide concentration was noted only with use of the usual brand cigarette, since there is no combustion during the use of Vuse Solo ECs or nicotine gum.

Subjects were not experienced EC users prior to enrollment, which may be seen as a potential limitation of the current study. Indeed, previous studies have shown that nicotine uptake from ECs may be dependent upon the level of experience with the products, and there are differences in puffing topography for experienced EC users versus smokers of combustible cigarettes (Farsalinos et al. 2013, 2015; Spindle et al. 2015). Based on those results, one might expect to see somewhat different puffing and inhalation patterns, which could lead to different —and perhaps higher—levels of nicotine uptake, and different subjective responses in experienced users. In order to somewhat alleviate the inexperience factor, subjects were provided Vuse Solo ECs to use for 6 days prior to the test visits to become familiar with them. However, as the intent was to assess abuse liability of these ECs in smokers, it was appropriate to exclude subjects who might have developed a strong preference or dislike for ECs based on recent use prior to study enrollment.

Conversely, a usual brand, combustible cigarette comparator was chosen in this study rather than a common comparator product in order to maximize the potential positive effects for the positive control condition. While it is understood that use of a usual brand comparator as a control could confound familiarity with nicotine delivery (Evans and Hoffman 2014), use of a control product that smokers do not like would presumably bias the positive control scores in a direction that would suggest weaker positive effects. The usual brand approach is consistent with previous studies that have evaluated subjective effects and nicotine uptake (Schuh et al. 1997; Stitzer and de Wit 1998; West et al. 2000; Houstsmuller et al. 2002; Vansickel et al. 2012b), and usual brand is more representative of the “real-world” scenario.

In summary, this study is the most robust assessment of the abuse liability of ECs published to date and uses approaches similar to those found in classic abuse liability studies of pharmaceutical products, including multiple instruments to measure the subjective effects of product use, as well as nicotine uptake. Under the set of study conditions described herein, use of the three Vuse Solo ECs tended to result in subjective measures responses and nicotine uptake that were between those measured with use of combustible cigarettes and nicotine gum. In general, the results are consistent with the conclusions of others that the abuse liability of ECs as a category is less than that of combustible cigarettes but greater than for nicotine gum, and likely other nicotine replacement products (Abrams and Niaura 2015; Schuh et al. 1997; Shiffman et al. 2000). These findings suggest that EC products such as Vuse Solo may have sufficient abuse liability to serve more effectively than NRT as a cigarette replacement for some smokers. However, the EC category is very diverse and continues to evolve, and therefore product differences will need to be considered in bridging study findings to other vapor products. Advances in product design and battery technology, coupled with varying nicotine levels and e-liquid flavors that may assist smokers to migrate away from combustible cigarettes, will certainly be expected to impact product use behaviors. Continued research will provide a better understanding of the category’s utility as an alternative to smoking combustible cigarettes and its potential to contribute to public health.

## Electronic supplementary material

ESM 1 (DOCX 31 kb)
