# Supplementary material for: Pharmacodynamic and pharmacokinetic assessment of electronic cigarettes, combustible cigarettes, and nicotine gum: implications for abuse liability
Source: Psychopharmacology (Berl). 2017 Jun 20;234(17):2643–55. doi: 10.1007/s00213-017-4665-y (PMC5548902; doi:10.1007/s00213-017-4665-y)
Supplement: Supplementary file 1 — (DOCX 31 kb) [file 213_2017_4665_MOESM1_ESM.docx]

**Supplementary Material File**

**Article Title:** Pharmacodynamic and pharmacokinetic assessment of electronic cigarettes, combustible cigarettes, and nicotine gum: implications for abuse liability

**Journal Name:**  *Psychopharmacology*

**Authors and Affiliations:** Mitchell F. Stiles^1^, Leanne R. Campbell^1^, Donald W. Graff^2^, Bobbette A. Jones^1^, Reginald V. Fant^3^, Jack E. Henningfield^3^

^1^RAI Services Company, Winston-Salem, NC, USA

^2^Celerion, Lincoln, NE, USA

^3^Pinney Associates, Inc., Bethesda, MD, USA

**Corresponding Author:** Mitchell F. Stiles; RAI Services Company; [stilesm@rjrt.com](mailto:stilesm@rjrt.com)

**Supplementary Table 1. Timeline of Test Visit Events**

| Study Event | Time^a^ | BL | | 3 | | 5 | | 7.5 | | 10 | | 15 | | 20 | | 30 | | 35 | | 45 | | 60 | | 75 | | 90 | | 120 | | 150 | | 180 | | 240 | | 300 | | 360 | |
| --- | --- | --- | --- | --- | --- | --- | --- | --- | --- | --- | --- | --- | --- | --- | --- | --- | --- | --- | --- | --- | --- | --- | --- | --- | --- | --- | --- | --- | --- | --- | --- | --- | --- | --- | --- | --- | --- | --- | --- |
| Product Liking | |  |  | |  | |  | |  | | X | |  | | X | |  | | X | | X | |  | |  | | X | |  | | X | | X | | X | | X | |  |
| Intent to Use Again | |  |  | |  | |  | |  | | X | |  | | X | |  | | X | | X | |  | |  | | X | |  | | X | | X | | X | | X | |  |
| Product Effects | |  |  | |  | |  | |  | | X | |  | | X | |  | | X | | X | |  | |  | | X | |  | | X | | X | | X | | X | |  |
| Urge to Smoke | | X |  | | X | |  | |  | | X | |  | | X | |  | | X | | X | |  | | X | | X | |  | | X | | X | | X | | X | |  |
| Urge for Product^b^ | | X |  | | X | |  | |  | | X | |  | | X | |  | | X | | X | |  | | X | | X | |  | | X | | X | | X | | X | |  |
| Blood Sampling | | X | X | | X | | X | | X | | X | | X | | X | |  | | X | | X | | X | | X | | X | | X | | X | | X | | X | | X | |  |
| Pulse Rate and Blood Pressure | | X |  | |  | |  | |  | | X | |  | | X | |  | | X | | X | |  | |  | | X | |  | | X | | X | | X | | X | |  |
| Expired Carbon Monoxide | |  |  | |  | |  | |  | |  | |  | |  | | X | |  | |  | |  | |  | |  | |  | |  | |  | |  | |  | |  |

BL=Baseline (baseline time points for blood sampling included -5 and -0.5 minutes relative to start of product use).

^a^Time (minutes) expressed relative to the start of product use.

^b^Urge for Product was administered only during VUSE Solo EC and nicotine gum Test Visits.

Supplementary Table 2. Mean Absolute Change in Pulse Rate and Blood Pressure from Baseline

|  |  | | | **VUSE Solo ECs vs  Usual Brand Cigarette** | | **VUSE Solo ECs vs  Nicotine Gum** | |
| --- | --- | --- | --- | --- | --- | --- | --- |
| **Parameter** | **LS Means of Maximum Absolute Change** | | | **Difference**  **(90% CI)** | **p-Value** | **Difference**  **(90% CI)** | **p-Value** |
| **Pulse Rate (bpm)** |  |  |  |  |  |  |  |
| VUSE Solo 14 mg | 13.85 | | | -1.73 | 0.1438 | -0.01 | 0.9927 |
|  |  |  |  | (-3.67 - 0.22) |  | (-1.95 - 1.93) |  |
| VUSE Solo 29 mg | 16.83 | | | 1.26 | 0.3011 | 2.97 | 0.0145 |
|  |  |  |  | (-0.75 - 3.26) |  | (0.98 - 4.96) |  |
| VUSE Solo 36 mg | 14.96 | | | -0.61 | 0.6099 | 1.10 | 0.3526 |
|  |  |  |  | (-2.60 - 1.37) |  | (-0.85 - 3.05) |  |
| Usual Brand Cigarette | 15.57 | | |  | | | |
| Nicotine Gum | 13.86 | | |  | | | |
| **Systolic Blood Pressure (mmHg)** | | | | | | | |
| VUSE Solo 14 mg | 16.51 | | | -1.60 | 0.2540 | -3.17 | 0.0232 |
|  |  |  |  | (-3.90 - 0.71) |  | (-5.46 - -0.88) |  |
| VUSE Solo 29 mg | 18.33 | | | 0.22 | 0.8782 | -1.36 | 0.3422 |
|  |  |  |  | (-2.16 - 2.60) |  | (-3.71 - 1.00) |  |
| VUSE Solo 36 mg | 17.01 | | | -1.10 | 0.4412 | -2.68 | 0.0583 |
|  |  |  |  | (-3.45 - 1.25) |  | (-5.00 - -0.35) |  |
| Usual Brand Cigarette | 18.11 | | |  | | | |
| Nicotine Gum | 19.69 | | |  | | | |
| **Diastolic Blood Pressure (mmHg)** | | | | | | | |
| VUSE Solo 14 mg | 11.81 | | | -4.12 | 0.0005 | -3.98 | 0.0007 |
|  |  |  |  | (-6.04 - -2.21) |  | (-5.89 - -2.08) |  |
| VUSE Solo 29 mg | 13.13 | | | -2.80 | 0.0200 | -2.66 | 0.0256 |
|  |  |  |  | (-4.77 - -0.83) |  | (-4.62 - -0.71) |  |
| VUSE Solo 36 mg | 13.16 | | | -2.77 | 0.0202 | -2.63 | 0.0252 |
|  |  |  |  | (-4.72 - -0.82) |  | (-4.56 - -0.70) |  |
| Usual Brand Cigarette | 15.93 | | |  |  |  |  |
| Nicotine Gum | 15.80 | | |  |  |  |  |
